# Language usage shapes neural mechanisms of code-switching

**DOI:** 10.3389/fpsyg.2026.1959633

**Published:** 2026-10-21

**Authors:** Stephen Theron-Grimaldi, Mirjana Bozic

**Affiliations:** Department of Psychology, University of Cambridge, Cambridge, United Kingdom

**Keywords:** alpha oscillations, attention control, bilingualism, code switching, EEG, language usage, neuroplasticity

## Abstract

Linguistic environments play an important role in shaping the mechanisms of language processing. The current study examined how neural responses to bilingual code-switching vary as a function of ongoing language usage, and which control processes may underlie these differences. Forty highly proficient English-French bilinguals listened to continuous spoken narratives containing inter-sentential switches. Participants were matched for proficiency and language background but differed in their current L2 usage (Active vs. Inactive). Narratives alternated either between their first and second languages, or between language and an acoustically matched non-linguistic signal, providing a means to probe language disengagement and re-engagement. While comprehension and cortical speech tracking were comparable across groups, oscillatory analyses revealed a usage-dependent modulation of alpha-band activity. Active bilinguals showed comparable alpha responses to switch and non-switch sentences, whereas Inactive bilinguals showed reduced alpha power following language switches, emerging from 650 ms over parieto-occipital regions. The direction, timing, and location of this effect are consistent with later attention control processes rather than early perceptual responses to the switch. Further analyses suggested that group differences were more closely associated with language disengagement than re-engagement. Together, these findings demonstrate an association between current L2 usage and alpha-band responses during language switching, suggesting that ongoing language experience may be modulating the neural mechanisms of language control.

## 1 Introduction

Neuroplasticity, the brain’s ability to change in response to experience, is a fundamental property of human cognition. This capacity for adaptation extends to complex cognitive functions, including the ability to learn and use more than one language: bilingualism. This study focuses on one of the hallmarks of bilingual language processing – the ability to alternate between languages—to ask how its neural underpinnings may adapt and change with ongoing language use.

### 1.1 Experience-dependent adaptations in bilingualism

Understanding and producing speech relies on distributed neural systems that extend beyond language-specific processes, from auditory perception to semantic retrieval (Bozic et al., 2010; Hickok and Poeppel, 2007). For bilinguals, this system is placed under additional demands, as two languages must be managed within the same cognitive architecture (Abutalebi and Green, 2007). As a result, using multiple languages relies not only on language knowledge itself, but also on cognitive control processes that help bilinguals select the intended language, manage interference from the other one, and alternate between them.

Consistent with this view, a growing number of studies have shown that bilingualism is associated with both structural and functional neural adaptations. For example, work comparing bilinguals to monolinguals has reported differences in grey matter volume or density in regions involved in language and domain-general control (Abutalebi et al., 2015; Mechelli et al., 2004), as well as changes in functional recruitment and connectivity across broader executive control networks (DeLuca et al., 2020; Sulpizio et al., 2020). Importantly, such adaptations are not limited to early development. Although neuroplasticity was once thought to be largely restricted to childhood, it is now widely understood as a lifelong process, with substantial evidence showing that adult experience can continue to reshape neural organisation (Draganski and May, 2008; Fuchs and Flügge, 2014). The same appears to hold for bilingualism, as adults can continue to acquire and refine a second language well beyond childhood (Gurunandan et al., 2019; Schlegel et al., 2012). Moreover, studies on lifelong bilinguals show different neural adaptations depending on how much they continue to use their languages across the lifespan (Luk et al., 2011; Olsen et al., 2015; Perani and Abutalebi, 2015). This means that neural adaptation likely reflects not only the initial acquisition of a second language, but also the continuing effects of language usage, practice, and changes in language experience and contexts over time (Gullifer et al., 2018; Hartanto and Yang, 2016).

The Adaptive Control Hypothesis (Green and Abutalebi, 2013) provides an influential account of this idea, by proposing that the control demands of bilingual language use are not fixed, but depend on how languages are used in everyday life. As a result, different patterns of bilingual experience may place different demands on control processes, which in turn may lead to cognitive and neural adaptations that change over time. This perspective also has important implications for how bilingualism should be studied. Although comparisons between bilinguals and monolinguals have revealed important neural differences, they provide only broad insight into which aspects of bilingual experience drive such adaptations. Because bilingualism is not a unitary condition, recent work has increasingly moved towards exploring cognitive processing within the bilingualism continuum, in order to identify how differences in bilingual language experience shape the underlying cognitive and neural processes (De Bruin, 2019; DeLuca et al., 2019; Salig et al., 2021).

### 1.2 Code-switching and the role of language experience

One particularly relevant domain for examining how language experience shapes bilingual processing is a phenomenon unique to bilingual language use: code-switching. Code-switching is defined as the ability of bilinguals to alternate between their languages. Although bilinguals often switch languages in everyday life with apparent ease, research has frequently reported poorer or slower performance in switch trials compared with non-switching trials (Meuter and Allport, 1999). This effect has been shown in language production and comprehension, and is also reflected in neural measures (Costa and Santesteban, 2004; Kaan et al., 2020; Olson, 2017). The so-called switch cost has emerged as a key marker in the study of code-switching, and is commonly used to investigate the cognitive control processes involved in managing two languages (for a review, see Declerck and Philipp, 2015). As a result, a large body of work has examined how switch costs vary across bilingual populations and across different dimensions of bilingual experience. A classical approach has been to investigate the influence of proficiency, which appears to shape the pattern of switching performance. Studies commonly report switch asymmetries between languages, with bilinguals who are not fully proficient in non-native language(s) showing larger differences when switching into one language than the other (Meuter and Allport, 1999), whereas highly proficient bilinguals often show reduced or absent asymmetry (Costa and Santesteban, 2004; Costa et al., 2006). However, proficiency does not appear to have a consistent effect on overall switch cost magnitude (Gade et al., 2021). This may be because proficiency is a relatively broad and stable characteristic, and therefore may not capture the more dynamic aspects of bilingual experience that shape code-switching adaptation over time. Converging evidence also comes from research into the effects of the age of acquisition (AoA), which is an inherently fixed factor and does not appear to be a strong predictor of switching performance (Bonfieni et al., 2019; Costa et al., 2006).

Thus, researchers have increasingly examined language experience as a key variable shaping bilingual adaptation in the code-switching literature. This has been approached from several complementary dimensions, with the simplest and most intuitive measure being switching behaviour. Some studies have examined individual differences in switching habits (Rodriguez-Fornells et al., 2012), showing that bilinguals who report switching between languages more frequently in daily life tend to exhibit smaller switch costs (Han et al., 2022), whereas less frequent switchers generally show bigger costs (Prior and Gollan, 2011). Another approach focused on how bilinguals use their languages across contexts. These studies examine the extent to which language usage varies or overlaps across social settings, and show that more diverse or overlapping language usage is associated with reduced switch costs (Hartanto and Yang, 2016) and enhanced neural connectivity in regions involved in language control (Gullifer et al., 2018). Despite this evidence however, various questions persist. For instance, several studies failed to find an association between switching habits and cognitive control performance (Jiao et al., 2019; Paap et al., 2017), while others diverge in the mechanisms through which language use across contexts influences control processes (with, e.g., Gullifer et al., 2018 discussing proactive control; and Hartanto and Yang, 2016 emphasising post-switch reconfiguration). A recent review also confirms that simple measures of language-switching frequency do not consistently predict cognitive control outcomes, whereas broader aspects of bilingual experience, such as overall language usage, may be more informative (Rayo et al., 2024).

Therefore, an alternative and broader way to assess language experience is to examine the overall usage of each language. This is typically measured as the proportion or frequency of use over time, and aims to capture the level of sustained engagement with each language (Anderson et al., 2018; Birdsong et al., 2012). Unlike switching behaviour or contextual diversity, overall usage directly reflects whether a language is actively maintained or progressively disengaged: for instance, a bilingual may report low switching frequency, yet still use both languages daily (e.g., one at home and another at work), thus maintaining the increased cognitive control demands. In contrast, a reduced overall usage of a language would reflect a diminished need to recruit and sustain control over that language. In this sense, global usage is not simply another proxy of switching, but a key measure of whether language control mechanisms remain continuously engaged (DeLuca et al., 2019). In addition to providing an arguably more direct and comprehensive way to capture bilingual experience, the overall language usage also allows for testing both the emergence and maintenance of bilingual adaptations, and how these adaptations may weaken when a language becomes less used. This is important, because a reduction in usage is not simply the absence of further learning, but may instead reveal whether previously acquired adaptations are still maintained. Recent studies are consistent with this idea, with structural brain measures suggesting that bilingual adaptations may reach a plateau with continued experience (Korenar et al., 2023) and decline when language is not in use (Hosoda et al., 2013; Tu et al., 2015). Moreover, behavioural and neuro-cognitive evidence indicates that only a strong reduction in usage, rather than relatively small differences in usage among active bilinguals, reveals changes in attention control (Theron-Grimaldi et al., 2025). This raises the possibility that the absence of clear effects in some code-switching studies may partly reflect a lack of sensitivity to variation in overall usage.

### 1.3 Control mechanisms underlying code-switching

Beyond establishing how changes in linguistic environment, as captured by usage, modulate switching, it remains equally important to understand the mechanisms that give rise to switch costs in the first place. Neurocognitive studies examining switch-related processing have most commonly used ERPs, with studies reporting effects in several components, including early positivities, late positive component (LPC), as well as N400 effects (Jiang et al., 2023; Moreno et al., 2002; Valdés Kroff et al., 2020). More recent work has also relied on changes in oscillatory power, with switch-related effects reported across several frequency bands, from slow Delta (1–4 Hz) to faster Theta (4–8 Hz) or Alpha (8–12 Hz) bands (Fernandez et al., 2019; Jiao et al., 2025; Litcofsky and Van Hell, 2017; Timofeeva et al., 2023; Tomić and Kaan, 2022). Functionally, Delta and Theta activity have been associated with speech and language processing, including the temporal encoding of continuous speech (Di Liberto et al., 2015; Ding et al., 2016; Doelling et al., 2014; Peelle et al., 2013), whereas Alpha activity has been more consistently linked to attention control and the regulation of relevant and irrelevant information (Klimesch, 2012; Obleser et al., 2012; Wöstmann et al., 2017; Dimitrijevic et al., 2017). The broad range of neural effects in code-switching likely arises because, in addition to switch costs, switching between languages engages multiple other operations that overlap in time, ranging from language-specific processes such as lexical competition and sentence-level integration, to control-related processes such the relative activation state of the languages and the dynamic shift in their activation at the moment of switching (Green, 1998; Philipp et al., 2007). As a result, observed switch effects are difficult to attribute to one specific process alone. Language-related aspects of switching have been examined widely, particularly in production, where switching has been linked to processes such as lexical selection, speech planning, and phonological or phonetic adjustment across languages (Beatty-Martínez et al., 2020; Olson, 2016). By contrast, the control-related component remains less well understood, particularly because multiple attentional mechanisms are engaged simultaneously and are difficult to isolate directly. More specifically, during a language switch, bilinguals must not only engage with the new target language, but also disengage from the previously used language, with several theoretical accounts proposed to explain the underlying mechanisms. According to the Inhibitory Control account, the non-target language is actively suppressed and therefore becomes harder to re-activate following a switch (Green, 1998). Alternatively, the Residual Activation account attributes switch costs to lingering activation of the previously used language (Philipp et al., 2007). Because these processes are inherently confounded during switching, it remains difficult to determine which mechanism primarily drives the observed switch cost. Classical language-switching tasks do not allow these processes to be cleanly dissociated, and some studies have used more targeted designs to address this issue. For instance, the N-2 repetition cost paradigm, usually using a picture naming task, isolates the consequences of inhibition by showing that returning to a recently used language is more difficult when that language was previously suppressed (Philipp et al., 2007). Other approaches have used bimodal designs, taking advantage of sign-speech bilinguals’ ability to produce both languages simultaneously and therefore separate turning one language on from turning the other off. These studies showed that the main cost of switching likely lies in disengaging from the previous language, rather than in engaging the new one, again supporting an important role for inhibition in switching performance (Blanco-Elorrieta et al., 2018; Emmorey et al., 2020).

Nevertheless, such paradigms have been primarily used in production and comparatively little is known about how these control processes unfold during speech comprehension, where switches must be detected and resolved in real time without explicit cues—even though bilinguals are evenly, if not more likely to encounter mixed-language input rather than to produce it (Fernandez et al., 2019; Guzzardo Tamargo et al., 2016; van Hell, 2022). Furthermore, within the existing comprehension literature, the dominant approach has been reading-based. This work has been highly informative for understanding bilingual language processing at the lexical and syntactic level (Beatty-Martínez and Dussias, 2017; Moreno et al., 2002; Wang, 2015), sometimes through the use of sensitive methods such as eye-tracking (Guzzardo Tamargo et al., 2016; Valdés Kroff et al., 2018). Yet, since readers can regulate the pace of processing by slowing down and rereading the input (Bultena et al., 2015; Faroqi-Shah and Wereley, 2022), studying code-switching through the visual modality may only provide limited insight into the control processes engaged at the moment of a language switch. Thus, while reading comprehension may be informative for investigating proactive control mechanisms (Jiang et al., 2023), it is likely less well suited to capturing the real-time control demands involved in adapting to a language switch once it occurs. Although some studies have reduced this issue with more constrained designs that limit preview of the switch (Adler et al., 2020), such tasks also move further away from natural code-switching comprehension, which may alter the very mechanisms they aim to capture. This issue is especially important given recent evidence that switch costs are shaped not only by bilingual experience, but also by the immediate linguistic context within the task itself. For instance, manipulating the relative presence of each language can directly modulate switching performance (Timmer et al., 2025), suggesting that variation in observed switch costs may arise from how languages are distributed within the task. In parallel, studies using targeted paradigms have also shown that language-related factors such as proficiency can modulate the control processes involved in switching (Babcock and Vallesi, 2015), highlighting the importance of carefully measuring bilingual experience when investigating switching mechanisms. Together, these findings indicate that while the control processes underlying switch costs remain difficult to isolate, the observed effects may vary both as a function of bilingual experience and of the specific paradigm used to measure them.

With all this in mind, a promising alternative is to study language switches in auditory comprehension, where bilinguals must detect and adapt to a change in language directly from the unfolding speech signal itself. Existing speech-comprehension studies already suggest that auditory code-switching can recruit control-related processes (Abutalebi et al., 2007), yet it remains rare to find naturalistic paradigms in the research on bilingual code-switching during speech comprehension, where the materials often remain highly controlled (e.g., relying on isolated words, short sentences, or tightly constrained task structures, Coumel et al., 2024; Fernandez et al., 2019; Gross et al., 2019), and speech comprehension tasks often come with additional auditory or multimodal cues (Fricke et al., 2016; Jiao et al., 2025; Tomić and Kaan, 2022) rather than requiring listeners to infer the switch from continuous speech alone. Although some recent work started to address this limitation by using more continuous auditory materials to closely replicate natural speech comprehension (Pérez and Duñabeitia, 2019; Salig et al., 2025), such approaches remain relatively uncommon in the literature.

Taken together, the existing evidence highlights three key challenges for research into bilingual code-switching. First, it remains unclear whether code-switching mechanisms in bilinguals flexibly track the current language usage, or persist as relatively stable consequences of past bilingual experience. Second, the control processes underlying switching behaviour remain difficult to isolate, as neural markers of switching remain uncertain and the specific control processes driving switch costs remain difficult to disentangle. Third, there is a need to investigate these issues in ecologically valid paradigms that capture switching more naturally, particularly in the speech comprehension domain.

### 1.4 Current study

The present EEG study examines the association between current language usage and code-switching in spoken language comprehension, and which control mechanisms support this process. We tested highly proficient English-French bilinguals who had all acquired French by age 11 and have achieved a high level of proficiency, but who differ in their current L2 usage. They were divided into two groups: those who use their L2 daily (Active users) and those who use their L2 only very sporadically (Inactive users). This comparison was intended to assess the role of ongoing language engagement. In addition to comparing these distinct usage profiles, we also modelled second-language usage continuously. In a naturalistic continuous listening paradigm, participants listened to spoken narratives with inter-sentential switches occurring every few sentences. We focused on inter-sentential switches because, unlike intra-sentential switches—which additionally require substantial lexical retrieval and within-sentence syntactic integration (Bultena et al., 2015; van Hell et al., 2015; Fernandez et al., 2019)—inter-sentential switches minimise these demands while retaining the need to disengage from the previously active language and engage with the new one, allowing us to examine attention control adaptation with less influence from within-sentence integration processes. The design included three conditions. The first condition involved switching between participants’ L1 and L2, in which narratives alternated between English and French. The other two conditions involved switching between either L1 or L2 and an acoustically matched non-linguistic signal called Musical Rain (MuR). Unlike switches between two languages, these transitions remove the presence of a competing language, allowing us to tap into processes involved in disengaging from language (e.g., L1 or L2 ➔MuR) and those involved in re-engaging with it (e.g., MuR ➔ L1 or L2). Using this design and comparing bilinguals based on current language usage, we address two key questions. First, in the language-switching condition (L1_L2), we tested whether current language usage is associated with differences in bilinguals’ processing of switches during continuous speech comprehension, which would indicate that this process may vary as a function of ongoing linguistic environment. Second, using the acoustic-switching conditions, we examined which control processes may contribute to these differences by probing language disengagement and re-engagement. By comparing these processes across groups, we determine whether differences in switching were more likely to reflect the demands of disengaging from the previous language or re-engaging with the new language.

To characterise the neural effects of switching, we focused on spectral analyses of the EEG signal, while also including behavioural and speech tracking analyses as complementary measures. Across previous studies, alpha-band modulations have emerged as a recurring neural signature of language switching (Jiao et al., 2025; Litcofsky and Van Hell, 2017; Timofeeva et al., 2023; Tomić and Kaan, 2022). We therefore focused on alpha power (8–12 Hz) given its established association with attention control and its potential relevance to the control demands involved in transitions between sentences. Although reduced alpha power for switch relative to non-switch trials has been reported in several studies (Jiao et al., 2025; Litcofsky and Van Hell, 2017; Timofeeva et al., 2023; Tomić and Kaan, 2022), both increases and decreases in alpha power have been reported across different attentional and listening demands (Obleser et al., 2012; Grant et al., 2022; Wisniewski et al., 2017), including across bilinguals differing in language usage (Theron-Grimaldi et al., 2025). We therefore interpret alpha modulation in the context of its direction, timing, spatial distribution, and task demands, rather than assuming a fixed functional interpretation of its direction.

In the language-switching condition (L1–L2), we examined whether this modulation differed as a function of language usage. Based on the comparable evidence from switching research (e.g., Han et al., 2022), we expected that Active bilinguals might show more stable response across switch and non-switch trials compared to Inactive bilinguals, reflecting differences in how control is deployed. Any effects were expected to emerge in a relatively late time window, consistent with previous work reporting switch-related activity only from 350–450 ms onwards (Fernandez et al., 2019; Litcofsky and Van Hell, 2017; Timofeeva et al., 2023; Tomić and Kaan, 2022). Given that both groups were highly proficient, we did not *a priori* expect asymmetries between switching into L1 and L2. In the acoustic-switching conditions, we aimed to dissociate the control processes underlying switching by comparing transitions from language to MuR (disengagement) with transitions from MuR to language (re-engagement). Based on previous studies (Blanco-Elorrieta et al., 2018; Emmorey et al., 2020; Philipp et al., 2007), we expected that any usage-group differences would emerge primarily during disengagement transitions. Finally, behavioural and cortical speech tracking analyses were included to ensure that any usage-related neural effects were not driven by differences in speech processing or global task engagement. Together, this design allows us to examine whether neural signatures of code-switching vary as a function of current language usage and to investigate the control processes that may contribute to these differences.

## 2 Materials and methods

### 2.1 Participants

Forty-two young adults studying or working at Cambridge took part in the experiment. They were all native speakers of English (L1) and fully proficient in French (L2); with some also occasionally using additional languages. All reported acquiring English before French. Two participants were excluded from the data analysis due to low comprehension accuracy (2σ below the average). The final sample size was therefore 40 participants (mean age: 20.9; 11 M, 29F), aged between 18–26 years old. None reported any hearing, attention or language disorders. The study was reviewed by the Psychology Research Ethics Committee of the University of Cambridge. All participants provided informed consent and were compensated for their participation.

### 2.2 Demographic questionnaire and L2 proficiency

All participants completed a questionnaire assessing demographic information and language background, as well as a French proficiency test. The questionnaire was derived from the Bilingual Language Profile questionnaire (Birdsong et al., 2012) and the Language and Social Background Questionnaire (Anderson et al., 2018). It included the frequency, intensity, and modality of language usage. The French proficiency test consisted of three 2-min audio recordings and 15 multiple-choice questions. The materials were taken from the highest level of the DELF (Elder, 2018), a French language qualification awarded by the French Ministry of Education. Although the standard pass threshold for this test is 50%, only individuals achieving a score of 80% or above were retained, ensuring that all included participants demonstrated a high level of French proficiency.

Based on the language background questionnaire, participants were classified into two groups depending on their frequency of L2 usage: Active-Usage (daily) and Inactive-Usage (fewer than once a week), and we also assessed the effects of Usage as a continuous variable in parallel. In addition to the questionnaire responses, usage was verified in person. A summary of demographic and linguistic characteristics for each group is provided in Table 1. The two groups were closely matched in terms of demographic variables (Sex, Age, Education level) and language experience (AoA, Proficiency and number of Languages Spoken).

**Table 1:** Bilinguals’ profile across usage groups.

Characteristics	Usage		Group comparison*^a^*
	Active *N = 20*	Inactive *N = 20*	
Demographic			
Sex (% male)	20%	35%	*p* = 0.294
Age	20.9 (2.1)	20.9 (1.6)	*p* = 0.945
Education level*^b^*	1.4 (0.7)	1.4 (0.5)	*p* = 0.698
Language			
L2 usage*^c^*	37.3%	1.8%	*p* < 0.001
L2 proficiency score	91.7 (5.8)	91.0 (5.4)	*p* = 0.739
L2 AoA	6.0 (4.7)	8.2 (4.4)	*p* = 0.102
Languages spoken*^d^*	2.8 (0.6)	2.6 (0.7)	*p* = 0.178

^a^Kruskal-Wallis test; ^b^1 = A-Levels, 2 = Undergraduate, 3 = Masters, 4 = PhD; ^c^Reported proportion of time spent using French and other non-native languages (French was dominant in all cases); ^d^Total number of languages spoken, including English (L1) and French (L2).

SD in brackets.

### 2.3 Experiment design and stimuli

We used a code-switching paradigm in which participants were presented with a continuous auditory stream that, depending on condition, alternated either between two languages or between a language and a non-linguistic signal called Musical Rain (MuR). The experiment consisted of three conditions: L1–L2 (in which the language of the narrative alternated between participants’ L1 English and L2 French), MuR–L1 (where the narrative was presented in English and alternated with MuR segments), and MuR–L2 (where the narrative was presented in French and alternated with MuR segments). In the L1–L2 condition, the narrative remained continuous across switches and, to minimise speaker-related differences (Brungart and Simpson, 2007), both languages were spoken by the same native bilingual speaker. In the MuR conditions, the narrative resumed when the stream returned to L1 or L2. MuR stimuli were created from natural speech by extracting their temporal envelopes and reconstructing them using 10 ms segments of synthesised speech that were randomly perturbed in both frequency and periodicity (Bozic et al., 2010). This procedure maintains low-level acoustic properties of speech while eliminating linguistic intelligibility. The MuR segments were derived from stories in the corresponding language, so that MuR–L1 contained English-based MuR and MuR–L2 contained French-based MuR, ensuring that low-level temporal properties between MuR and speech were matched and remained constant throughout listening. All switches were inter-sentential and occurred after one, two, or three sentences. The overall number of switching and non-switching sentences was matched. The relatively high proportion of switch trials ensured a sufficient number of observations for the EEG analyses, although it may have increased participants’ expectation that a switch could occur. This was mitigated by the stimulus recording procedure described below, which minimised acoustic and prosodic cues signalling an upcoming switch.

Each condition included four blocks of a continuous auditory stream. In the L1–L2 condition, each block contained an equal number of English and French sentences, resulting in a narrative of 60 sentences in total per block. In the MuR–L1 and MuR–L2 conditions, each block also contained 60 sentences, with a narrative of 30 sentences in the corresponding language and 30 sentences’ worth of MuR. Children’s stories were used as narrative material. Each story comprised 120 sentences in total. Therefore, two stories were used in the L1–L2 condition, and one story was used in each of the MuR–L1 and MuR–L2 conditions. Sentences were recorded individually, edited in Audacity (version 3.2.3, 2022) to last approximately 3 s, with a 300 ms silence gap inserted between sentence before concatenation. This approach intentionally reduced naturalistic acoustic and prosodic cues across sentence boundaries that could otherwise signal an upcoming language switch (e.g., Fricke et al., 2016), thereby minimising proactive anticipation of the switch and allowing us to focus on participants’ responses to the language change itself. This procedure resulted in blocks lasting approximately 3 min 15 s each. All stories were normalised to the same root mean square sound amplitude.

### 2.4 Procedure

Participants first completed a short practice trial to familiarise themselves with the task. They were then presented with 12 listening blocks, each followed by comprehension questions. The first condition presented was always L1-L2, so participants could familiarise themselves with the switching paradigm. We counterbalanced which of the two stories within that condition was presented first. Next, we also counterbalanced between the other two conditions, so either MuR-L1or MuR-L2 was presented first. To ensure that participants were paying attention to the narrative, they were told they will be completing a set of 10 comprehension questions after each block. This resulted in a total of 120 comprehension responses per participant. Comprehension questions were presented in L1 in the MuR-L1 condition and in L2 in the MuR-L2 condition, matching the language of the narrative. In the L1–L2 condition, questions were presented in L1 to minimise additional language-switching demand at the response stage. Participants answered the comprehension questions on a laptop. No feedback was provided. The experiment took approximately 1 h and 15 min.

### 2.5 Behavioural data

The comprehension task consisted of 40 multiple-choice questions per condition, with four answer options for each question. Additionally, participants were required to indicate their confidence in each answer by stating whether they were certain, unsure, or guessing. Six questions were removed from the analysis, due to their difficulty (average response accuracy lower than 50%), retaining 114 answers per participant. Comprehension accuracy was modelled using Generalised Linear Mixed-Effects Models in RStudio (version 4.2.2, 2022). The data were fitted with the fixed effects of Usage Group (Active, Inactive), Condition (L1–L2, MuR-L1 and MuR-L2), and the control variables of L2 AoA, L2 Proficiency, and number of Languages Spoken. Random intercepts for Participants and Questions were included. For the parallel analyses of continuous Usage effects, Usage Group was replaced by the variable Continuous Usage. For this analysis, self-reported usage scores were log transformed to compress differences between them, especially at the upper end, thus placing greater emphasis on proportional differences in current usage. Contrasts were dummy coded, with the L1-L2 condition as the reference level, so that the acoustic-switching conditions (MuR-L1 and MuR-L2) could be evaluated relative to the language-switching baseline. *p*-values were calculated using the Type III sum of squares method with the Satterthwaite approximation for degrees of freedom, provided by the Anova function of the “car” package (version 3.1–2, Fox & Weisberg, 2019). Post-hoc tests were performed using the “emmeans” package (version 1.8.6, Lenth et al., 2023) applying a Tukey correction. Significant *p*-values are reported at the 0.05 threshold.

### 2.6 EEG data acquisition and pre-processing

A high-density 128 channel electrodes cap (Electrical Geodesics Inc., Eugene, OR, USA) was connected to the EGI Net-Station software (version 5.4.2) via a GES 400 amplifier. Impedance for each electrode was kept under 100 Ω. 36 channels located in the peripheral areas of the net were excluded to reduce muscle artifacts. The data were preprocessed in MATLAB using the EEGLAB toolbox (Delorme and Makeig, 2004; version 2024.2). A bandpass filter from 1 to 40 Hz was applied to the continuous data, which was subsequently down-sampled to 100 Hz. Bad channels were identified using automatic detection based on kurtosis (±5 SDs) and power spectrum (±3 SDs); all flagged channels were visually inspected to verify that they had been correctly identified. After interpolation of the bad channels and re-referencing to the average of all channels, an Independent Component Analysis (ICA) was performed for artefact detection. ICA components were automatically classified using the ICLabel plugin (Pion-Tonachini et al., 2019). A component was flagged as brain-related if its ICLabel brain probability was at least 25%, allowing mixed components with partial neural contribution to be retained for further evaluation. A visual inspection was then performed to confirm or adjust the rejection decisions, with particular focus on the first 24 components for each participant, from which 39.8% (SD = 17.8) were rejected.

Listening data were epoched into 3 s listening segments, from −200 ms relative to sentence onset to 2,800 ms post-onset. We also extracted baseline data for power analyses, from a 10s pre-listening period in each block, segmented into matching 3 s epochs. The first listening epoch of each block was excluded to account for potential attentional settling effects. Bad epochs were automatically rejected using the pop_autorej tool, applying amplitude and joint-probability thresholds (±3 SD), resulting in a final rejection rate of 10.76% for listening epochs and 3.06% for baseline epochs, consistent with recommended standards (Delorme et al., 2007).

### 2.7 Power spectral density analysis

Power Spectral Density (PSD) analysis was performed to quantify oscillatory activity during listening, using the FieldTrip toolbox (Oostenveld et al., 2011) in MATLAB. Power was baseline-corrected by subtracting resting-state power from listening-period power within each condition. Global power was computed across the full listening epoch (0–2.8 s) using the multitaper frequency transformation method (mtmfft) with a Hanning taper, in 0.5 Hz steps from 1 to 12 Hz. Band power was calculated as the mean power within different frequency ranges: Delta (1–4 Hz), Theta (4–8 Hz), and Alpha (8–12 Hz). However, subsequent statistical comparisons of experimental effects focused on Alpha, as Delta and Theta did not show reliable modulation relative to baseline (see Results). We compared Switch with NoSwitch trials within each condition separately, with the linear mixed-effects models in each condition testing for the effects of Switch Type (Switch vs. NoSwitch), Usage (either categorical and continuous) and Language that participants listened (English vs. French). As in the behavioural analyses, post-hoc tests were conducted using the “emmeans” package with a Tukey correction for multiple comparisons. To examine temporal dynamics, we also computed time-resolved alpha power (8–12 Hz), using a sliding-window time–frequency approach (mtmconvol) with a Hanning taper, estimated every 50 ms across the epoch (−0.2 to 2.8 s). Because power estimates are unreliable at the epoch boundaries, the supported time window was 0.05 to 2.55 s. Based on the spatial distribution observed in the topographical maps and the existing evidence on likely distribution of alpha-related effects (Theron-Grimaldi et al., 2025; Klimesch, 2012; Wöstmann et al., 2017; Dimitrijevic et al., 2017; Foxe and Snyder, 2011), time-resolved analyses focused on parieto-occipital regions. We performed cluster-based permutation tests implemented in FieldTrip (ft_freqstatistics) with a dependent-samples t-statistic and Monte Carlo randomisation of 1,000 permutations. Tests were two-tailed with cluster correction, using a cluster-forming threshold of *p* = 0.05 and maxsum as the cluster statistic. Finally, Pearson correlations were conducted to investigate the relationship between individual alpha switch-related effects in L1–L2 condition and power changes in the MuR conditions.

### 2.8 mTRF analysis

The multivariate Temporal Response Function (mTRF) analysis captures continuous and stimulus-specific tracking and was conducted using the mTRF Toolbox in MATLAB (Crosse et al., 2016) to measure neural tracking of the speech envelope across different types of signals (L1, L2 or MuR). Switch Type was not included because code-switching reflects transient changes in processing state that are better suited to the power analyses described above. Speech envelopes were extracted using the ‘Hilbert2’ function from EEGLAB and downsampled to 100 Hz to match the EEG sampling rate. EEG data were z-score normalised and bandpass filtered between 1 and 12 Hz to attenuate high-frequency noise. Epochs from the preprocessed EEG data were concatenated for each participant and condition, with 100 ms of zero-padding between epochs to prevent edge artefacts. The same procedure was applied to the corresponding speech envelopes.

Backward (decoding) mTRF analysis was used to estimate how accurately the speech envelope could be reconstructed from the EEG signal. Models were computed on approximately 10 min of continuous listening data using a 10-fold cross-validation procedure, with a time-lag window from 0 to 600 ms. The resulting Pearson correlation coefficients (r-values) between the predicted and actual speech envelopes were averaged across folds. R-values were obtained for each Participant, Condition and Signal Type (L1, L2 or MuR where applicable), and entered into linear mixed-effects regression models (lmer) in R to assess effects of Usage (categorical and continuous), Condition (L1–L2, MuR-L1 and MuR-L2) and Signal Type (L1, L2 or MuR where applicable). As before, post-hoc comparisons were conducted with a Tukey correction for multiple comparisons, and the *p*-values were considered significant at *p* < 0.05.

To estimate chance-level performance against which model performance could be evaluated, a permutation approach was used in which the speech envelope was circularly shifted by a randomised lag, disrupting the temporal alignment between stimulus and response while preserving the overall acoustic structure. 100 r-values were generated from permuted data, resulting in 600 control r-values per participant across the three conditions and their corresponding signal types. Using paired-sample t-tests, we confirmed that observed r-values were significantly higher than their respective control values in all conditions and signal types (Appendix, Supplementary Table A4), confirming reliable neural tracking above chance level. Following Crosse et al. (2021), we also calculated d-prime scores by comparing the actual *r*-values to those from the permuted models for each participant. The average d-prime was 2.14 (SD = 0.63), indicating robust model performance well above the commonly used reliability threshold.

## 3 Results

### 3.1 Behavioural analysis

We first aimed to establish whether the bilinguals’ language usage modulates comprehension of the narratives across the switching conditions. To that end, we ran a model including the fixed effects of Usage Group (Active, Inactive), Condition (L1–L2, MuR–L1, MuR–L2), their interaction, and the control variables of L2 AoA, L2 Proficiency and Languages spoken. Results (Supplementary Table A1a in the Appendix) showed no significant effects (Group [*χ*^2^(1) = 0.02, *p* = 0.890]; Condition [*χ*^2^(2) = 3.42, *p* = 0.181] or Group*Condition interaction [*χ*^2^(2) = 4.72, *p* = 0.095], with the analyses of the continuous Usage effects replicating all these findings (Supplementary Table A1b). Overall, participants answered the questions with an average accuracy of 88.2% (SD = 5.5) (Table 2, Supplementary Figure A1), and reported guessing their answers only 3.6% (SD = 3.5) of the time, indicating that they understood the stories well and were able to perform the task effectively.

**Table 2:** Comprehension scores and standard deviation by condition and group.

Condition	Active	Inactive
L1-L2	89.0% (3.1)	89.1% (3.1)
MuR-L1	90.7% (2.9)	88.6% (3.2)
MuR-L2	88.3% (3.2)	83.3% (3.7)

### 3.2 Power spectral density analyses

Power Spectral Density analyses were used to assess switching-related neural oscillatory responses in the two usage groups. As an initial validation step, we examined whether oscillatory power during listening in each group differed from resting-state baseline within Delta, Theta, and Alpha frequency bands. Results showed that power did not significantly differ from baseline in the Delta band [Active: *t*(59) = 0.35, *p* = 0.729; Inactive: *t*(59) = −0.05, *p* = 0.962] or the Theta band [Active: *t*(59) = 1.58, *p* = 0.121; Inactive: t(59) = 1.50, *p* = 0.138]. In contrast, Alpha power significantly increased during listening in both Active [*t*(59) = 2.77, *p* = 0.008] and Inactive bilinguals [*t*(59) = 4.74, *p* < 0.001]. We then also established that this Alpha band increase was not driven by a specific condition in either group [Active: *F*(2, 38) = 0.14, *p* = 0.868, η^2^ = 0.00; Inactive: *F*(2, 38) = 0.17, *p* = 0.845, η^2^ = 0.00] indicating comparable levels of task engagement and attentional demand across the board.

#### 3.2.1 Usage and code-switching

In line with the key question of this study, we next turned to examining Alpha power activity across different code-switching conditions, and whether this might be associated with language usage. This was first investigated in the L1–L2 language-switching condition. To do so, alpha power was analysed as a function of Usage Group (Active, Inactive), Switch Type (Switch vs. NoSwitch sentences), Language (English, French), and their interactions (Figure 1A, Supplementary Table A2a). Results showed no main effects of either Usage Group [*F*(1, 38) = 0.08, *p* = 0.783, η^2^ = 0.00], Switch Type [*F*(1, 114) = 1.66, *p* = 0.201, η^2^ = 0.01] or Language [*F*(1, 114) = 0.84, *p* = 0.360, η^2^ = 0.00]. However, there was a significant interaction between Usage Group and Switch Type [*F*(1, 114) = 7.54, *p* = 0.007, η^2^ = 0.06], with the same results also emerging from the analyses of continuous Usage effects (Supplementary Table A2b). Post-hoc comparisons showed that there were no differences in alpha power between switch and non-switch sentences in Active bilinguals [*t*(114) = −1.03, *p* = 0.305], but that there was a reduction in alpha power for switch relative to non-switch sentences in Inactive bilinguals [*t*(114) = 2.85, *p* = 0.005] (Figure 1B). There was no evidence for any interaction involving Language (Supplementary Table A2), indicating that the switch effect in Inactive bilinguals was not driven by English or French specifically. In other words, there was no evidence of switch asymmetry, and the switching effect was present in both directions (L1 → L2 and L2 → L1).

**Figure 1:**
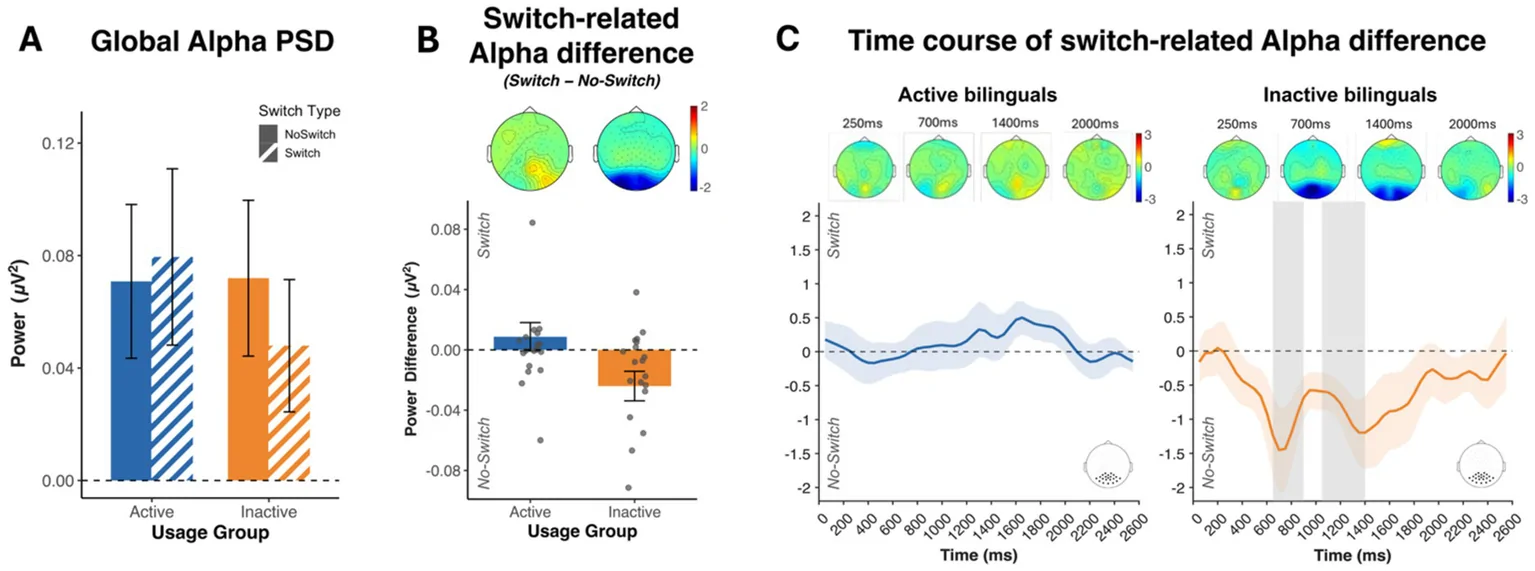
Alpha-band modulation during language switching. **(A)** Global Alpha Power Spectral Density (PSD), showing a reduction in baseline-corrected alpha power during switch compared to non-switch sentences in Inactive bilinguals. **(B)** Switch-related alpha difference (Switch—No-Switch), highlighting the switch effect in Inactive bilinguals and its posterior scalp distribution. **(C)** Time course of the switch-related alpha difference, showing alpha power differences over time (0–2,600 ms) in the parieto-occipital region, with no significant switch effect observed in Active bilinguals, but showing a significant difference from 650 to 1,400 ms in Inactive bilinguals (as indicated by the grey window). Error bars and shaded areas represent ± SEM.

A set of follow-up analyses examined spatial distribution and temporal dynamics of the observed reduction in alpha power for switch sentences in Inactive bilinguals. This revealed that the effect originated from parietal and occipital sites (Figure 1B), in line with the literature on alpha power during tasks recruiting attention and control mechanisms (Klimesch, 2012; Wöstmann et al., 2017; Dimitrijevic et al., 2017; Foxe and Snyder, 2011). Applying an exploratory time-resolved analysis to the observed parieto-occipital cluster showed that in Inactive bilinguals, the alpha decrease on switch trials was present from around 350 ms, but only became statistically reliable from 650 to 1,400 ms, before progressively returning towards non-switch levels (Figure 1C). These findings suggest that the switch effect in Inactive bilinguals emerged relatively late, rather than as an immediate response to the switch, suggesting that the observed alpha modulation was less consistent with early sensory processing, but more likely related to later attention control processes involved in resolving the switch between languages.

#### 3.2.2 Mechanisms of code-switching

We next turned our attention to the acoustic-switching conditions (MuR–L1 and MuR–L2) to test whether the observed code-switching effects were more likely related to disengagement or re-engagement processes (see Figure 2A for an overview of the task comparisons). Because MuR–L1 and MuR–L2 were designed to capture the same type of transition between linguistic and non-linguistic input, and because there were no differences between L1 and L2 in oscillatory power in the previous analysis, data from these two conditions were analysed together.

**Figure 2:**
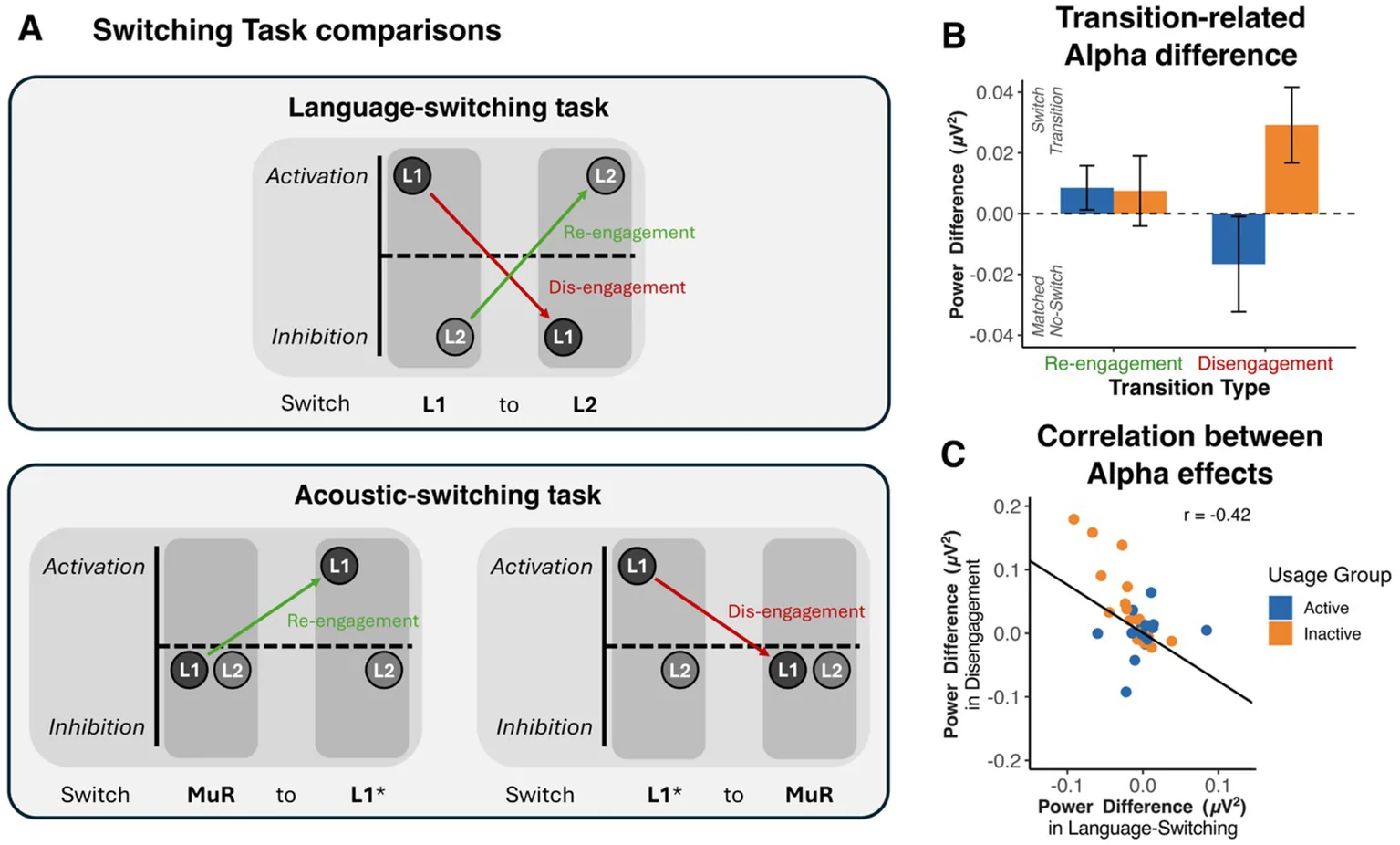
Dissociating language disengagement and re-engagement during switching. **(A)** Switching task comparisons. The top panel illustrates how the language-switching task involves simultaneous processes of language re-engagement and disengagement, using the example of an L1 → L2 switch that includes both the re-activation of L2 and the inhibition of L1 (equivalent processes occur during L2 → L1 switches). Bottom panel illustrates how the acoustic-switching task isolates each process, using the example of a Musical Rain (MuR) → L1 switch involving the re-activation of L1, reflecting language re-engagement, and an L1 → Musical Rain (MuR) switch involving the de-activation of L1, reflecting language disengagement (equivalent processes occur during switches involving L2). **(B)** Transition-related alpha difference between switch and matched non-switch transitions, showing alpha power differences between Active and Inactive bilinguals during disengagement transitions. **(C)** Correlation between alpha power differences in language switching and disengagement transitions, showing a negative relationship between the switch-related alpha effect and the disengagement-related alpha effect across participants. Error bars represent ± SEM.

Switching from language to MuR was used to probe language-disengagement and switching from MuR to language was used to probe language-re-engagement, although these transitions also involve the broader stopping and initiation of linguistic processing and are therefore not pure measures of disengagement or re-engagement. To avoid confounding switching effects with inherent differences between linguistic and non-linguistic signal, each switch type was first assessed against a matched no-switch baseline. Disengagement was therefore calculated as the difference in alpha power between language→MuR switches and language→language no-switch trials (L1 → MuR vs. L1 → L1; L2 → MuR vs. L2 → L2). This contrast indexed the effect of interrupting ongoing language processing. Re-engagement was calculated as the difference in alpha power between MuR → language switches and MuR → MuR no-switch trials (MuR → L1 and MuR → L2 vs. MuR → MuR). This contrast indexed the effect of returning to language processing from a non-linguistic context. The resulting alpha power was then analysed as a function of Usage Group, Transition Type (disengagement, re-engagement), Language, and their interactions. This analysis revealed no main effect of Usage Group [*F*(1, 38) = 2.43, *p* = 0.127, η^2^ = 0.06], Transition Type [*F*(1, 114) = 0.02, *p* = 0.882, η^2^ = 0.00], or Language [*F*(1, 114) = 0.43, *p* = 0.512, η^2^ = 0.00]. However, it showed a significant Usage Group by Transition Type interaction [*F*(1, 114) = 4.20, *p* = 0.043, η^2^ = 0.04; Figure 2B, Supplementary Table A3a], with these results again replicated in the analyses of continuous Usage effects (Supplementary Table A3b). Post-hoc comparisons indicated that this interaction was driven by disengagement, which revealed a significant group difference [*t*(114) = −2.50, *p* = 0.014], with Inactive bilinguals showing an increase in alpha power, and Active bilinguals showing a slight negative modulation. By contrast, no group difference emerged for re-engagement [*t*(114) = 0.06, *p* = 0.955], with alpha power remaining close to zero in both groups and indicating that they responded similarly when switching from MuR to language. Similarly to in the L1-L2 analysis, we found no evidence for any interaction involving Language (Supplementary Table A3). This again indicates that the observed group difference was not specific to either English or French, but instead reflects a more general usage effects on disengagement from the linguistic signal.

#### 3.2.3 Relationship between language-switch and disengagement-related modulations

Finally, to test whether the language-switch modulations in alpha power observed in the L1-L2 condition (Section 3.2.1, Figure 1B) were associated with alpha power during disengagement-related effects observed in the analyses of acoustic-switching conditions above, we conducted an exploratory Pearson correlation across participants. To this end we correlated language-switch modulation for each participant (calculated as the difference in alpha power between switch and non-switch L1/L2 sentences) with their disengagement-related modulation in the MuR conditions (calculated as the difference between language-to-MuR transitions and matched no-switch language trials).

This analysis therefore tested whether participants showing a larger alpha modulation during language switching also tended to show a stronger alpha modulation during disengagement. It revealed a significant negative correlation (*r* = −0.42, *p* = 0.006, p_*_Bonferroni_* = 0.018), indicating that participants who showed a larger increase in alpha power during disengagement also tended to show a larger decrease in alpha power during language switching (Figure 2C). To further determine whether this relationship could be explained by between-group differences, we ran a follow-up regression analysis predicting the language-switch effect from both the disengagement effect and Usage Group. Results confirmed that the disengagement-related effect remained a significant predictor of the language-switch effect after accounting for Usage Group [*F*(1, 37) = 8.79, *p* = 0.005, η^2^ = 0.19], whereas Usage Group itself was not significant [*F*(1, 37) = 3.13, *p* = 0.085, η^2^ = 0.08]. This indicates that the association between the two alpha effects was not solely attributable to differences between Usage Groups, with individual variation in disengagement associated with variation in the language-switch effect.

Although the two alpha effects were expressed in opposite directions, their significant relationship suggests that they may reflect related attention control processes engaged differently across transition contexts. More specifically, this raises the possibility that individual differences in language-switch modulation may be linked to differences in disengagement-related modulation, an interpretation which we return to in the Discussion.

### 3.3 Cortical speech tracking

Backward mTRF decoding was used to quantify the cortical tracking of speech envelopes across switching conditions and usage groups. Overall, the tracking of the envelope was robust with no differences across Condition [*F*(2, 196) = 1.26, *p* = 0.287, η^2^ = 0.01] or Usage, modelled either categorically [*F*(1, 38) = 1.75, *p* = 0.193, η^2^ = 0.04] or continuously [*F*(1, 38) = 2.93, *p* = 0.095, η^2^ = 0.07]. This indicates that bilinguals, regardless of their current L2 usage, encoded the acoustic signal to a similar degree across switching contexts (see Supplementary Tables A5a,b in the Appendix for full results). We further examined whether tracking differed between signal types (L1 and/or L2 and/or MuR, depending on condition) within each condition. In the L1–L2 condition, tracking did not differ between English and French speech [*F*(1, 38) = 1.67, *p* = 0.205, η^2^ = 0.04], suggesting comparable envelope encoding of L1 and L2 during continuous narrative processing. However, in both the MuR–L1 and MuR–L2 conditions, speech was more strongly decoded than the non-linguistic signal [MuR–L1: *F*(1, 38) = 9.63, *p* = 0.004, η^2^ = 0.20; MuR–L2: *F*(1, 38) = 37.63, *p* < 0.001, η^2^ = 0.50], with the analysis of continuous usage effects again confirming these results. See Supplementary Figure A2 and Supplementary Tables A6a,b in the Appendix for full results. Together, these findings demonstrate that cortical tracking was sensitive to whether the input was linguistic or non-linguistic, but was not modulated by language usage or switching condition.

## 4 Discussion

The present study investigated whether bilinguals’ neural responses to language switching during speech comprehension vary as a function of their current language usage, and whether any such differences are more closely associated with processes tapping into disengaging from or re-engaging with language. We tested highly proficient English–French bilinguals who were matched for L2 proficiency and learning history, but strongly differed in their frequency of L2 usage. Usage was modelled both categorically and continuously, and all effects replicated across the two types of analyses. In the language-switching condition (L1–L2), Inactive bilinguals showed reduced alpha power during switch relative to no-switch trials, whereas Active bilinguals showed no difference between the two types of trials. This suggests that switch-related modulations of alpha activity in bilinguals emerge specifically when current usage of the non-native language is low. In the acoustic-switching conditions (MuR–L1 and MuR–L2), we observed usage-group differences in alpha power during language-to-MuR transitions, which provide a measure of the neural processes associated with disengagement from ongoing language processing. By contrast, no group difference was observed during MuR-to-language transitions, suggesting that usage is not associated with processes involved in re-engagement into language. Further exploratory analyses revealed a significant association between this disengagement-related alpha effect and the switch-related modulation of alpha activity in the language-switching condition. Finally, our switching tasks did not trigger significant changes in Delta or Theta power relative to baseline. Moreover, we found no usage-related differences in the cortical tracking of speech or in behavioural comprehension of the switching narratives. Jointly, these results suggest that the effects observed in the current study are more consistent with differences in attention control associated with current language usage, rather than with differences in language processing per se.

### 4.1 Language usage modulates the neural control of code-switching

We used power spectral density analyses to capture the neural mechanisms engaged during language switching, with a particular focus on alpha power. At first, we confirmed that performing our code-switching comprehension task was associated with an overall increase in alpha power relative to baseline. This effect fits well with the broader literature linking alpha activity to the deployment of attention under demanding listening conditions (Klimesch, 2012; Tune et al., 2021), particularly during active speech listening and as listening demands increase (Dimitrijevic et al., 2017; Obleser et al., 2012; Wöstmann et al., 2017). This likely reflects the sustained attentional engagement required to follow the ongoing narrative and maintain task-relevant information, compared with the resting-state baseline. We then asked whether this alpha response changes as a function of language switching, and whether this might be associated with language usage. The results showed a clear difference between switch and no-switch sentences, but only in Inactive bilinguals. Indeed, in active bilinguals, alpha power remained similarly high in switch and no-switch sentences. By contrast, inactive bilinguals showed reduced alpha power on switch sentences relative to no-switch sentences. This suggests that current language usage may be modulating the neural demands associated with switching between languages. The observed switch effect is consistent with the existing code-switching literature, in which alpha power has emerged as one of the most reliable oscillatory markers of switch-related processing (Fernandez et al., 2019; Jiao et al., 2025; Litcofsky and Van Hell, 2017; Timofeeva et al., 2023; Tomić and Kaan, 2022). However, the nature of this modulation has varied across studies, with most reporting reduced alpha power during switching (Jiao et al., 2025; Timofeeva et al., 2023; Tomić and Kaan, 2022), but some also finding increases or effects that vary as a function of switch direction (Fernandez et al., 2019; Litcofsky and Van Hell, 2017). The alpha-power reduction observed here is therefore consistent with one commonly reported pattern, although the absence of a switch-direction asymmetry contrasts with some previous findings, which we discuss further below.

Functioanlly, alpha modulation has been widely associated with attention control and has been shown to vary as a function of task demands (Klimesch, 2012; Tune et al., 2021; Wöstmann et al., 2017), although it is not uniquely specific to attention control or inhibition. More specifically, reduced alpha power has often been linked to increased processing demands, including more effortful listening and stronger recruitment of control mechanisms under challenging conditions (Grant et al., 2022; Obleser et al., 2012; Theron-Grimaldi et al., 2025; Timofeeva et al., 2023; Wisniewski et al., 2017). In this context, the reduced alpha power observed on switch sentences in Inactive bilinguals suggests that language switching was likely more demanding in this group. However, although the observed alpha switch effect is consistent with increased attention control demands, an alternative possibility is that it could reflect differences triggered by the change in input language. We therefore tested this possibility directly, by assessing how our listeners neurally encode sentences in their L1 and L2, as well as their behavioural comprehension across the two languages. Our results show that neither analysis supported this account. At the neural level, we found no evidence that Inactive bilinguals differed from Active bilinguals in cortical tracking of speech. At the behavioural level, the two groups also performed similarly, with both showing good overall comprehension of the stories. Together, these findings suggest that the observed group difference is unlikely to be driven by differences in language processing. This interpretation is further supported by the frequency-specific analyses. Among the frequency bands examined, only Alpha band showed reliable modulation relative to baseline during the switching task, whereas Delta and Theta bands did not. The absence of modulation in these lower-frequency bands should nevertheless be interpreted with some caution, particularly for Delta, as the relatively short epoch duration may have limited the reliability of the power estimates. This pattern is nonetheless informative, given that slower frequency bands are more commonly associated with aspects of speech and language processing (Di Liberto et al., 2015; Ding et al., 2016; Doelling et al., 2014; Peelle et al., 2013). The specificity of the observed modulations to the Alpha band is therefore consistent with the interpteration of the switching-related effect in terms of attention control demands rather than language processing specifically.

Another finding that contributes to the interpretation of the current effects is that the switch effect observed in Inactive bilinguals was not modulated by switch direction. In other words, the reduction in alpha power was present for both L1-to-L2 and L2-to-L1 transitions, rather than being driven by one direction only. This absence of asymmetry is consistent with previous work showing that switch costs are not necessarily direction-dependent. This appears to be particularly the case in bilinguals with high proficiency (Costa and Santesteban, 2004; Costa et al., 2006), and in comprehension paradigms (Thomas and Allport, 2000; Macizo et al., 2012). Nevertheless, the literature remains mixed regarding when and why such asymmetries may arise. This variability is also present in studies measuring oscillatory power. On one hand, some work has reported direction-dependent alpha effects, even in highly proficient bilinguals and in comprehension paradigms (Fernandez et al., 2019; Litcofsky and Van Hell, 2017). On the other hand, researchers have reported symmetrical alpha decreases during switching, which they interpreted as a marker of language-independent control (Timofeeva et al., 2023). Asymmetry therefore remains incompletely understood, but importantly, it does not appear to be a necessary feature of switch-related neural costs. In the present study, the absence of direction effects suggests that the switch cost observed in Inactive bilinguals was not tied to one language being especially difficult to activate or process, but instead reflected a more general control demand arising whenever the input language changed.

By examining alpha power over time in the exploratory analysis over the parieto-occipital regions, we found that the switch effect in Inactive bilinguals emerged relatively late. More specifically, alpha power appeared lower for switch than no-switch sentences from around 350 ms after switch onset, but this effect became statistically reliable only between 650 and 1,400 ms. While these results should be interpreted cautiously given the data-informed selection of parieto-occipital ROI for time-resolved analyses, their timing is consistent with previous work showing that switch-related alpha modulations generally emerge after 350 ms (Timofeeva et al., 2023; Fernandez et al., 2019; Litcofsky and Van Hell, 2017), and can sometimes extend beyond one second (Tomić and Kaan, 2022; Jiao et al., 2025). They are also in line with ERP comprehension studies, which have often linked switching to late positive components (Liao and Chan, 2016; Ruigendijk et al., 2016; Fernandez et al., 2019; Yacovone et al., 2021). The timing of our effects thus further suggests that they are unlikely to reflect early perceptual or sensory processes, and points to a later process involved in resolving the switch instead. The posterior localisation of the effect additionally informs the interpretation of this later switch-related process. Indeed, alpha modulations over parieto-occipital regions are widely associated with attention control and the regulation of sensory processing during demanding listening situations (Klimesch, 2012; Wöstmann et al., 2017; Dimitrijevic et al., 2017; Foxe and Snyder, 2011). In the present case, the topography is consistent with the switch effect reflecting a control process operating on the incoming speech signal, rather than a more abstract operation detached from perception. In language switching, such a pattern is consistent with the possibility that the change in language modulates how attentional resources are allocated to the new input, plausibly through a gating-like mechanism (Jensen and Mazaheri, 2010; Wöstmann et al., 2017). This suggests that the observed switch effect may reflect activation and/or inhibition control processes, corresponding to re-engagement with the new language and disengagement from the previous one.

### 4.2 Language usage is primarily associated with disengagement

To better understand which control processes may underlie the language switch modulation, we turned to the acoustic-switching conditions. Unlike in the language-switching condition, these transitions did not require bilinguals to switch directly between two competing languages. Instead, by using the acoustic control MuR, we could tap into transitions from language processing (language→MuR) and transitions into it (MuR → language), providing a means of probing the control processes involved in disengaging from and re-engaging with language. The results suggest that usage-related differences were more closely linked to disengagement rather than re-engagement. Indeed, we found a group effect in alpha power specifically for language-to-MuR transitions, whereas no group difference was observed for MuR-to-language transitions. This suggests that current language usage is not associated with how bilinguals generally handle all transitions, but more likely how they disengage from an active linguistic state. Further analyses showed that this disengagement effect was not modulated by language, indicating that the pattern was comparable for L1 → MuR and L2 → MuR transitions. This again suggests that the effect was not tied to one language specifically, and is therefore consistent with the general switch effect previously observed in Inactive bilinguals in the L1-L2 condition.

The valence of the disengagement effect is also informative. Inactive bilinguals showed increased alpha power relative to matched no-switch language trials, while alpha power did not differ in Active bilinguals. Although this may initially appear inconsistent with the reduced alpha power observed during language switching in Inactive bilinguals, the two patterns arise in different contexts and likely reflect different expressions of the same broader control system. In the language-switching condition, listeners had to maintain attention to the unfolding narrative while adapting to a change in language, and reduced alpha power was consistent with a more effortful and less stable control state under continuous speech processing. By contrast, in the acoustic-switching condition, listeners did not have to continue processing linguistic input through the transition itself, nor did they have to deal with direct competition between two languages. Instead, the task required listeners to disengage from an ongoing linguistic state or re-engage with it. The relevant demand was therefore not the continuous competition and balancing between two languages, but arguably the control involved in engaging and disengaging language processing itself. Within the broader attention literature, increases in alpha power in relatively simple attention tasks have often been linked to gating and inhibitory processes, particularly when currently active input must be suppressed. The larger alpha response observed here can therefore be interpreted as consistent with greater control demands during disengagement, potentially involving inhibitory processes. Crucially, this does not imply that disengagement was more efficient in Inactive bilinguals, but rather that greater control was required to achieve it. By contrast, in re-engagement (MuR → language) transitions, groups showed no difference in alpha power which remained close to zero. This suggests that re-engaging with language from a non-linguistic context did not require substantial effort in either Active or Inactive bilinguals, at least as captured by the present alpha measure. Importantly, the alpha modulations found during language-switch and disengagement should also be distinguished from the overall increase in alpha power observed during the task. The latter may reflect global and sustained attentional engagement across the task, whereas the more specific within-task effects likely reflect transient modulations within this ongoing attentional state.

Having observed the switch cost effects in the language-switching condition and the disengagement-related effect in the acoustic-switching conditions, we also aimed to establish whether they may reflect related processes. To test this directly, we examined the association between alpha modulation during disengagement transitions in the MuR–L1 and MuR–L2 conditions, and alpha modulation during switching in the L1-L2 condition. The two measures were significantly negatively correlated: participants who showed a larger alpha power increase during language disengagement also tended to show a larger alpha power decrease during language switching. This relationship suggests that the two effects were not independent, but may reflect related control processes that are expressed differently across the two task contexts. Although the acoustic-switching paradigm does not fully reproduce language-switching, it was designed to tap into specific control processes involved with disengaging and re-engaging with language processing, which are normally difficult to disentangle in traditional language-switching tasks. Switching to MuR necessarily interrupts continuous linguistic processing and reduces direct competition between the two languages, meaning that the task does not include all the demands present in the L1-L2 condition. At the same time, this simplification may also be what makes the paradigm informative. By separating transitions from and back into language processing, it allows one component of switch-related control to be probed more directly. The significant association with the switch effect in language-switching further suggests that the disengagement-related effect is unlikely to be specific to the MuR task alone, but may instead reflect mechanisms also involved in language-switching itself. However, it should be noted again that the MuR transitions cannot fully separate disengagement from the broader stopping and restarting of linguistic processing, limiting the interpretation of this effect. A further limitation concerns condition order, as the L1–L2 condition was always presented first to familiarise participants with switching, meaning that cross-condition differences could partly reflect fatigue, habituation, or adaptation across the experiment. Thus, the acoustic-switching paradigm provides a partial but nonetheless useful window onto one of the control processes that is likely to contribute to switch cost.

At a theoretical level, our findings are compatible with accounts that place inhibition at the centre of bilingual language control, such as the Inhibitory Control account (Green, 1998). At the same time, the Residual Activation account (Philipp et al., 2007) cannot be ruled out, as lingering activation of the previous language may also increase the demands associated with disengagement. Our results also converge with evidence from other paradigms designed to isolate switching components, including N-2 repetition cost studies and bimodal switching designs, which likewise suggest that an important part of switch cost lies in disengaging from the prior language rather than engaging to the next one (Philipp et al., 2007; Blanco-Elorrieta et al., 2018; Emmorey et al., 2020). The present study extends this idea to continuous auditory comprehension and further suggests that the efficiency of these disengagement-related control processes is modulated by current language usage.

### 4.3 Current language usage contributes to bilingual adaptation to code-switching

Overall, the present findings suggest that adaptation to code-switching is not simply a fixed consequence of having learned two languages, but remains sensitive to how those languages are currently used. Although all participants were highly proficient bilinguals with similar language histories, switch-related alpha modulation differed as a function of current L2 usage. More specifically, a neural switch modulation was observed only in bilinguals who no longer used their L2 regularly, whereas no such effect was found in those who continued to use it daily. Importantly, our analyses suggest that this usage-related change did not reflect language processing itself, but more likely reflected differences in attention control. The acoustic-switching paradigm was particularly useful here, as it helped pinpoint disengagement from an active linguistic state as a likely key process affected by current language usage.

Our results thus indicate that the control mechanisms supporting code-switching remain at least partially experience-dependent, and that their efficiency may weaken when engagement with the non-native language becomes rare. This point is important for the broader bilingualism literature, because code-switching has often been examined in relation to relatively stable variables such as proficiency or AoA. The present results suggest that these factors may not be sufficient to explain variation in switch-related processing on their own. Even when proficiency is high and learning history is comparable, current language usage still appears to matter. Therefore, our study supports a more dynamic view of bilingual adaptation, in which the neural mechanisms involved in language control are not only shaped by past experience, but also maintained or weakened as a function of ongoing usage.

The present findings also highlight the importance of how bilingual experience is measured when studying code-switching. Rather than focusing on specific switching habits, we examined a broader measure of current language usage, capturing the extent to which the non-native language remains actively engaged in everyday life. This turned out to be particularly informative because Active bilinguals showed no neural switch cost at all. This suggests that variation in how often bilinguals switch between languages (e.g., switching habit) may not be the most sensitive way to explain switch-related control demands, particularly if switching is no longer costly in the first place. The fact that a switch cost emerged only in bilinguals with very low current L2 usage therefore suggests that broad language engagement, rather than switching frequency alone, may be especially relevant for understanding when switch-related control costs remain visible and when they no longer do. More broadly, these findings may also help to clarify why switch costs can vary substantially across studies. If current language usage affects how demanding switching is, then differences in how much bilinguals still use their two languages may help explain why switch costs are found in some bilingual groups but not in others, even when they appear similar on standard background measures. The effects observed in this study should not necessarily be generalised across bilingual populations however, given that we selected a relatively homogeneous sample of bilinguals to help control variability in language background and focus on current usage as the main variable of interest. Although the groups were closely matched on measured language-background variables, other unmeasured differences between them cannot be fully excluded. Future studies should therefore examine whether usage-related modulation of attention control during language switching is also observed across more diverse bilingual populations and linguistic contexts, including those differing in proficiency, language combination, age, sociolinguistic environment, or switching practices. Moreover, longitudinal studies examining changes in L2 usage over time could also provide more direct evidence for the association observed here between current language usage and neural switching dynamics.

Finally, the present study extends this perspective to a more naturalistic form of auditory comprehension. Much of the literature on bilingual adaptation has relied on production tasks (but see Fernandez et al., 2019) or highly controlled paradigms. Here, the effect of usage emerged during continuous speech comprehension, in a context that more closely resembles everyday listening. This suggests that bilingual adaptation exists across processing contexts, from explicit task performance to processing of naturalistic speech as it unfolds. Overall, the findings support a dynamic view of bilingual language control, in which current language usage continues to modulate the mechanisms involved in code-switching.

## Data Availability

The datasets presented in this study can be found at: https://osf.io/x6m4g.
