# Supplementary material for: Language usage shapes neural mechanisms of code-switching
Source: Front Psychol. 2026 Oct 21;17:1959633. doi: 10.3389/fpsyg.2026.1959633 (PMC13731376; doi:10.3389/fpsyg.2026.1959633)
Supplement: Supplementary file 1 [file Supplementary_file_1.DOCX]

**Appendix**

**Table A-1: Behavioural comprehension accuracy**

*Comprehension Accuracy ~ Condition*Usage_Group + AoA + Proficiency + Languages_Spoken + (1 | Participant) + (1 | Question)*

| Effect | Df | Chisq | Pr(>Chisq) | Sig |
| --- | --- | --- | --- | --- |
| (Intercept) | 1 | 140.53 | .000 | *** |
| Condition | 2 | 3.42 | .181 |  |
| Usage_Group | 1 | 0.02 | .890 |  |
| AoA | 1 | 0.07 | .788 |  |
| Proficiency | 1 | 1.79 | .180 |  |
| Languages_Spoken | 1 | 0.24 | .624 |  |
| Condition * Usage_Group | 2 | 4.72 | .095 |  |

**Figure A-1.** Behavioural performance across conditions. Comprehension accuracy showed no difference in comprehension performance across the L1–L2, MuR–L1, and MuR–L2 conditions and in Active and Inactive bilinguals. Error bars represent ±95% CI.

**Table A-2: Oscillatory power in the Alpha band, L1–L2 condition**

*R_value ~ Usage_Group*Switch_Type*Language + (1 | Participant)*

| Effect | NumDF | DenDF | Sum Sq | Mean Sq | F value | Pr(>F) | Sig | η² |
| --- | --- | --- | --- | --- | --- | --- | --- | --- |
| Usage_Group | 1 | 38 | 0.00 | 0.00 | 0.08 | .783 |  | .00 |
| Switch_Type | 1 | 114 | 0.00 | 0.00 | 1.66 | .201 |  | .01 |
| Language | 1 | 114 | 0.00 | 0.00 | 0.84 | .360 |  | .00 |
| Usage_Group * Switch_Type | 1 | 114 | 0.01 | 0.01 | 7.54 | .007 | ** | .06 |
| Usage_Group * Language | 1 | 114 | 0.00 | 0.00 | 0.16 | .685 |  | .00 |
| Switch_Type * Language | 1 | 114 | 0.00 | 0.00 | 0.10 | .758 |  | .00 |
| Usage_Group * Switch_Type * Language | 1 | 114 | 0.00 | 0.00 | 0.41 | .521 |  | .00 |

**Table A-3: Oscillatory power in the Alpha band, MuR conditions**

*R_value ~ Usage_Group*Transition_Type*Language + (1 | Participant)*

| Effect | NumDF | DenDF | Sum Sq | Mean Sq | F value | Pr(>F) | Sig | η² |
| --- | --- | --- | --- | --- | --- | --- | --- | --- |
| Usage_Group | 1 | 38 | 0.01 | 0.01 | 2.43 | .127 |  | .06 |
| Transition_Type | 1 | 114 | 0.00 | 0.00 | 0.02 | .882 |  | .00 |
| Language | 1 | 114 | 0.00 | 0.00 | 0.43 | .512 |  | .00 |
| Usage_Group* Transition_Type | 1 | 114 | 0.02 | 0.02 | 4.20 | .043 | * | .04 |
| Usage_Group* Language | 1 | 114 | 0.00 | 0.00 | 0.60 | .441 |  | .00 |
| Transition_Type * Language | 1 | 114 | 0.00 | 0.00 | 0.16 | .686 |  | .00 |
| Usage_Group * Transition_Type * Language | 1 | 114 | 0.00 | 0.00 | 0.10 | .748 |  | .00 |

**Table A-4: Speech decoding mTRF models tested against chance**

| Signal Type | Condition | | |
| --- | --- | --- | --- |
|  | *L1–L2* | *MuR–L1* | *MuR–L2* |
| English | t(39) = 10.62 *** | t(39) = 13.22 *** | / |
| French | t(39) = 13.48 *** | / | t(39) = 14.52 *** |
| Musical Rain | / | t(39) = 10.96 *** | t(39) = 15.83 *** |

*( *** p<.001; ** p<.01; * p<.05)*

**Table A-5: Speech decoding mTRF models across conditions**

*R_value ~ Condition*Usage_Group + (1 | Participant)*

| Effect | NumDF | DenDF | Sum Sq | Mean Sq | F value | Pr(>F) | Sig | η² |
| --- | --- | --- | --- | --- | --- | --- | --- | --- |
| Condition | 2 | 196 | 0.00 | 0.00 | 1.26 | .287 |  | .01 |
| Usage_Group | 1 | 38 | 0.00 | 0.00 | 1.75 | .193 |  | .04 |
| Condition * Usage_Group | 2 | 196 | 0.00 | 0.00 | 0.19 | .826 |  | .00 |

**Table A-6: Speech decoding mTRF models by Signal Type in each condition**

*R_value ~ Usage_Group*Signal_Type + (1 | Participant)*

| Effect | NumDF | DenDF | Sum Sq | Mean Sq | F value | Pr(>F) | Sig | η² |
| --- | --- | --- | --- | --- | --- | --- | --- | --- |
| L1–L2 | | | | | | | | |
| Usage_Group | 1 | 38 | 0.00 | 0.00 | 0.49 | .488 |  | .01 |
| Signal_Type | 1 | 38 | 0.00 | 0.00 | 1.67 | .205 |  | .04 |
| Usage_Group * Signal_Type | 1 | 38 | 0.00 | 0.00 | 0.46 | .502 |  | .01 |
| MuR–L1 |  |  |  |  |  |  |  |  |
| Usage_Group | 1 | 38 | 0.00 | 0.00 | 0.65 | .425 |  | .02 |
| Signal_Type | 1 | 38 | 0.01 | 0.01 | 9.63 | .004 | ** | .20 |
| Usage_Group * Signal_Type | 1 | 38 | 0.00 | 0.00 | 0.81 | .374 |  | .02 |
| MuR–L2 |  |  |  |  |  |  |  |  |
| Usage_Group | 1 | 38 | 0.00 | 0.00 | 2.13 | .152 |  | .05 |
| Signal_Type | 1 | 38 | 0.02 | 0.02 | 37.63 | .000 | *** | .50 |
| Usage_Group * Signal_Type | 1 | 38 | 0.00 | 0.00 | 1.29 | .263 |  | .03 |

**Figure A-2.** Cortical tracking within conditions. **(A) mTRF decoding of speech tracking (1–12 Hz)**, showing no differences between Active and Inactive bilinguals, but stronger tracking of language than Musical Rain (MuR) in the MuR–L1 and MuR–L2 conditions. Error bars represent ± SEM.
